# Targeting breast cancer outcomes‐what about the primary relatives?

**DOI:** 10.1002/mgg3.286

**Published:** 2017-05-31

**Authors:** Alison Johnston, Michael Sugrue

**Affiliations:** ^1^ Breast Centre North West Letterkenny University Hospital Letterkenny Ireland; ^2^ Donegal Clinical Research Academy Donegal Ireland

**Keywords:** Breast cancer, breast cancer detection, breast outcomes, breast screening, primary relatives

## Abstract

**Background:**

Up to 65% of newly diagnosed breast cancer patients had not been screened correctly before diagnosis resulting in increased stage of cancer at presentation. This study assessed whether their primary relatives are, in turn, assessed appropriately.

**Methods:**

An ethically approved prospective study involving 274 primary relatives of women diagnosed with breast cancer, between 2009–2012, at a symptomatic breast unit in Ireland. Telephone interview established: demographics, menstrual history, family history verification, breast screening history. Personal risk level was calculated and whether current screening met screening guidelines. Participants were enrolled into appropriate screening programs if currently not in one and results analyzed.

**Results:**

Two hundred and fifteen of the 280 (76.8%) newly diagnosed patients responded giving details of their 274 primary relatives; this made up the study cohort. Mean age 50 ± 10 (35–75). Thirty two percent were low risk, 64% moderate and 4% high. 190/274 (69%) were being screened appropriately. Seventy five relatives were then assessed with: mammography in 55, Mg and US in 16. Four underwent a biopsy and to date none had cancer. Surveillance was: annual screening in 48%; national screening program and General Practitioner (GP) in 33%; GP only in over 65s in 13%; 6% await further assessment at specialist genetics clinics where their surveillance will be decided.

**Conclusions:**

This study has identified an opportunity to improve the delivery of appropriate screening to higher risk primary relatives of patients with breast cancer. This necessitates an integrated national approach involving providers of primary care, patients and screening breast programs.

## Background

Governments globally have advocated screening for certain common cancers, in particular breast and bowel cancer, to allow earlier detection and improve outcome. This process has been tailored to those in the highest risk age group; usually in the 50–70 year olds. This screening is costly and discussions regarding over diagnosis are common (Rashidian et al. 2013; Arrospide et al. 2016; Rafia et al. 2016; Swain et al. 2016). Attempts have been made to tailor care to individuals rather than blanket cohorts of the population (Evans et al. 2012, 2014). Family history is a very important assessment tool in detecting those at risk (Welch et al. 2015). We know that primary relatives have at least twice the risk of certain cancers; especially breast cancer (Colditz et al. 2012).

While there are opponents of breast screening, concerned mainly regarding over diagnosis particularly in DCIS, we have identified in our region that women meeting breast screening or family history risk criteria who were not appropriately screened with mammography presented with more significant advanced stages of breast cancer (Johnston et al. 2015). Diagnosis in early stage of breast cancer offers major survival advantage and lesser forms of treatment, with five‐year relative survival rates of stage 1 approaching 100% compared to 93% for stage 11, 72% stage 111 and 22% stage 1V (American Cancer Society, 2016).

With an increased risk of breast cancer in particular for sisters, daughters and mothers of an affected primary relative it makes intuitive sense to tailor risk assessment. In the Direct Observation of Primary Care Study data from 4454 directly observed visits to 138 family physicians in Northeast Ohio was analyzed (Gotler et al. 2001). Family history was sought in only half of the new patients and less than a quarter of returning patients. In general primary relatives should commence screening a minimum of 5 years younger than the affected relative and at the age of 40 if in the moderate risk group (USPSTF, 2009, NHS Scotland, [Link]). Few studies, however, have looked at the uptake of screening by primary relatives.

This study assessed whether the primary relatives of breast cancer patients were being assessed appropriately in lieu of their increased risk of breast cancer.

## Methods

An ethically approved review of all 321 women diagnosed with breast cancer at Breast Centre North West, Letterkenny University Hospital between 2009 and 2012 was undertaken. 280/321 eligible women (41 deceased) were contacted by letter and requested to offer the details of their primary female relatives between the ages of 35–70. Demographics, stage at diagnosis, tumor grade and triple negative disease incidence were documented in this cohort.

All their primary relatives were then contacted with a view to enrolment into this ethically approved prospective study of their breast care. Following receipt of written consent from willing primary relatives a structured telephone interview was carried out to establish the participants’ demographics, menstrual history, family history verification and breast screening history.

Two additional questions were asked at the interview of the primary relative: (1) if you visited a General Practitioner (GP) since turning aged 35, for any health reason, was a family history recorded? (2) If you visited a GP since turning aged 35, for any health reason, was a clinical breast examination (CBE) performed?

Using the information collected at the interview National Institute for Health and Clinical Excellence (2006) and IBIS Breast Cancer risk evaluation Tool V6 (2004) were used to assess each individual's personal breast cancer risk level. Participants were stratified into one of three risk groups‐ population risk, moderate risk and high risk‐ as defined in National Institute for Health and Clinical Excellence (2006).

An assessment was then carried out to determine whether the participant's current screening regimen concurred with international screening guidelines relevant to their risk group. The criteria used to determine whether optimal screening and risk assessment practices were currently being delivered are shown in Table 1. An invitation to enrol into guideline driven personal screening programs was offered to those not currently in one.

**Table 1:** Risk levels and optimal screening criteria used in this study

Age group	Risk level^a^
**Biennial screening**	
Ages 50–65 years	Population or moderate‐risk groups
Age 40–49	Moderate‐risk group
Aged <40 with a first degree relative diagnosed with breast cancer before the age of 40	Commencing 5 years prior to the age of their relative's age at diagnosis
**Annual screening**	
Any age	High‐risk groups

a Risk levels defined using NICE CG 41 (2006).

The screening modalities utilized, results and follow‐up screening plans were analyzed. Breast screening modalities commonly used included mammography (Mg), ultrasound and ultrasound guided biopsy. Mammograms were classified and reported using the UK 5‐point breast imaging classification (Maxwell et al. 2009).

Data was expressed as mean and standard deviation for normally distributed data and medians and inter quartile range for non‐normal data.

## Results

215/280 (76.8%) of newly diagnosed breast cancer patients responded, their mean age was 61 ± 15 (29–92). 221 breast cancers were diagnosed in 215 women. Their stage at diagnosis, tumor grade and triple negative disease incidence is shown in Table 2. These women supplied contact details of 329 of their primary relatives and 274/329 (83.3%) of these accepted the invitation and made up the study cohort. 274 women, mean age 50 ± 10 (35–75) were studied; 32% were in the population risk group, 64% moderate and 4% high. 190/274 (69%) were currently receiving guideline driven breast screening relevant to their risk group and 84/274 (31%) were not.

**Table 2:** TNM Classification (Tumor, Node, Metastasis) of breast cancer patients who responded with information about their first degree primary relatives

TNM Classification		*n*	%
Tumor	T0	22	10.3
	T1	83	38.6
	T2	79	36.7
	T3	17	7.9
	T4	14	6.5
Nodes	N0	131	60.9
	N1	52	24.3
	N2	16	7.4
	N3	16	7.4
Metastasis	M0	203	94.4
	M1	11	5.1
	Mx	1	0.5
Stage	‐is	23	10.7
	1	64	29.8
	11	80	37.2
	111	36	16.7
	1V	12	5.6
Invasive tumor grade (*n* = 192)	1	26	13.6
	2	107	55.7
	3	59	30.7
Invasive triple negative disease		12	6.3

75/84 (89.3%) proceeded to have breast screening performed and 9/84 (10.7%) declined. Modes of assessment included mammography alone in 55/75 (73.3%), with ultrasound in 16/75 (21.3%) and with image guided biopsy in 4/75 (5.4%).

31/72 (43.1%) were classified on imaging as R1, 39/72 (54.2%) R2 and 2/72 (2.8%) R3. Three had radiological classification data missing, no breast cancers were detected in this index round of breast screening in *n* = 75.

In line with current international practice surveillance breast screening was planned annually in 48% and biennial in 33%. Six percent await further assessment at specialist genetics clinics where their surveillance will be decided and 13% have no planned surveillance.

When attending their GP 26% of participants had their family history recorded, 6% of GPs were already aware of their family history and in 68% no family history was recorded. A CBE was performed in 52% of participants; 39% when they were asymptomatic and 13% when they consulted their GP with breast related symptoms. Forty eight percent did not have a CBE.

## Discussion

This study identified over 2/3 of primary relatives of symptomatic breast cancer patients were being screened appropriately. The participants in this study are self‐selected and introducing some bias; they are very interested in their risk of breast cancer with 69% already engaged in appropriate breast screening. Walker et al. (2013) found that perceived risk of breast cancer appears to have a weak to moderate positive relationship with guideline mammography adherence among women with familial breast cancer risk. Worry about breast cancer risk has been reported as a barrier to mammography uptake by all women not only those with a family history of breast cancer (Andersen et al. 2003).

Our population cohort size is small, however, the region is rather unique with only a single hospital providing symptomatic breast care services; this facilitated invitation of primary relatives into our study. The exceptions are the primary relatives of the 41/321 (12.8%) of patients who had deceased within 4 years of diagnosis; this is comparable to the reported 12.5% in the unstandardized 4 year survival rates found in breast cancer patients in Ireland from 2006–2012 (NCRI, 2016). The initial response rate of 76.8%, from our women diagnosed with breast cancer, to the mailed survey inviting their primary relatives to take part in this study was high despite not being incentivized. Kelly et al. (2010) had a 71% response rate from breast cancer patients to their incentivized postal survey.

To evaluate family history risk and need for assessment we used a combination of both NICE criteria and the Tyrer‐Cuzick IBIS Risk Evaluation Tool version 6 (IBIS 2004, NICE, 2006). NICE provides a modest easy to use classification but has restrictions by not taking into account biological, hormonal and metabolic criteria built into the Tyrer‐Cuzick IBIS Risk Evaluation.

Sixty eight percent of our primary relatives were in the moderate or higher risk groups. Evans et al. (2012) risk assessed 8824 women attending a family history evaluation and screening program and 86.4% were at a moderate or higher risk level. Livaudais‐Toman et al. (2015) found 75% of the women, in their randomized controlled trial to evaluate a breast cancer risk education intervention, were at population risk for developing breast cancer.

The guidelines for breast screening vary internationally. The American Cancer Society makes strong recommendations for annual screening for ages 45–54 then biennial until life expectancy is less than 10 years (Oeffinger et al. 2015). British guidelines suggest every 3 years from ages 50–70 with an on‐going trial extension rolling out to include ages 47–73 (ISRCTN, 2009). A number of other countries have expanded their program to include 40 year olds and a significant number extending to 69; some even extending to ≤74 years (Altobelli and Lattanzi 2014). Nationally within Ireland the National Cancer Control Program recommends biennial mammography between 50 and 64; currently being extended to include women up to age 69 (NCSS, 2015). International screening criteria for those between 40 and 50 have been controversial and have changed over the years. In the absence of global gold standards in optimal screening the definitions adopted in this study were chosen as they were considered balanced and reasonable.

While 69% of our women were being screened according to International guidelines the care to 31% can be improved upon. NICE CG164 (2013) recommends that healthcare professionals should respond to a person who presents with concerns but should not, in most instances, actively seek to identify people with a family history of breast cancer (last updated 2004). A large study of both primary and secondary care medical professionals highlighted strong views against this non proactive methodology (Harris et al. 2011). Our study shows a more proactive campaign to advance care of women at higher risk is needed. Evans et al. (2012) showed that it is feasible to undertake detailed individual breast cancer risk evaluation within a national screening program and 95% of participants are happy to know their risk level and importantly they are willing to act on the information given.

No breast cancers were detected in this index round of breast screening, however, two patients had a cancer diagnosis at subsequent screenings; one invasive and one in situ. The natural history of DCIS is unclear but is thought by many to be a cancer precursor although the proportion of DCIS which progress to invasive is again unclear (Swain et al. 2016). Duffy et al. (2016) analyzed the screening data of over 5 million women within a National Breast Screening Program to determine the association between screen‐detected DCIS and subsequent invasive interval cancers. Their findings suggest that detection and treatment of DCIS is worthwhile in prevention of future invasive disease.

In line with international guidelines the younger, at moderate risk, participants in our study are now enrolled in annual mammography surveillance until they reach 50 years of age. Increasing evidence has seen a paradigm shift from recommending biennial to annual screening in women age 40–49 years with a moderate risk of breast cancer (NICE, 2013; Altobelli and Lattanzi 2014; Moss et al. 2015; Oeffinger et al. 2015).

Primary relatives were asked if their GP had taken a family history record and whether a CBE had been performed. Family health history (FHH) is a great tool to help personalize disease prevention in multifactorial diseases not only breast cancer; heart disease (Mulders et al. 2016), colorectal cancer (Lowery et al. 2016) and asthma (Guttmacher et al. 2004). The full potential of FHH is only obtained when taken in advance of disease presentation and the information acted upon in a proactive and appropriate fashion. Shared responsibility for communicating about FHH is crucial but is not without difficulties. Simple, at‐the‐visit, family history prompts geared to improve GPs ability to identify patients at high risk for six common conditions were unsuccessful (Zazove et al. 2015). Despite multiple USA national strategies initiated to improve individuals’ family history recording little change has occurred over a 10 year period; only 36.9% actively collecting it (Welch et al. 2015).

Controversy remains with respect to the efficacy of CBE as a screening tool. No RCTs have been performed. There is inadequate evidence to say that CBE reduces breast cancer mortality, however, there is sufficient evidence to say that it shifts the stage distribution of tumors detected toward a lower stage (Lauby‐Secretan et al. 2015). Due to the low risk of CBE and potential benefit, both the ACS and NCCN recommend yearly CBE in addition to screening mammograms (American Cancer Society, 2014; NCCN, 2014). CBE is a low cost tool advocated widely in low‐resource and middle‐ resource countries (Anderson et al. 2011; Gelband et al. 2016). Particularly for women less than 50 and more than 69 years of age, in the moderate‐ and high‐risk groups, it is recommended that CBE should be a part of routine periodic examinations by trained healthcare professionals (Provencher et al. 2016) and in older women (Jatoi 2015; Schwab et al. 2015).

General Practitioners appear to be missing proactive opportunities in regards to recording family history and performing CBEs. GPs had knowledge of the individual's family history in only 32%. This is much lower than that reported in the 2007 New South Wales Population Health Study where 64.9% had discussed their FHH with their GP (Dunlop et al. 2010).

This study has identified that there is an opportunity to improve the delivery of appropriate screening to higher risk primary relatives of patients with breast cancer. This necessitates an integrated national approach involving providers of primary care, patients themselves and screening breast programs.

## Conflict of Interest

All authors have indicated they have no potential conflicts of interest to disclose.
